# High Frequency Jet Ventilation during Initial Management, Stabilization, and Transport of Newborn Infants with Congenital Diaphragmatic Hernia: A Case Series

**DOI:** 10.1155/2013/937871

**Published:** 2013-01-02

**Authors:** Qianshen Zhang, Jason Macartney, Lita Sampaio, Karel O'Brien

**Affiliations:** ^1^Neonatal Intensive Care Unit, Maternal & Child Healthcare Hospital of Shenzhen City, Guangdong 518028, China; ^2^Department of Paediatrics, Mount Sinai Hospital, University of Toronto, Toronto, ON, Canada; ^3^Department of Respiratory Therapy, The Hospital for Sick Children, Toronto, ON, Canada; ^4^Department of Respiratory Therapy, Mount Sinai Hospital, Toronto, ON, Canada

## Abstract

*Objective*. To review experience of the transport and stabilization of infants with CDH who were treated with high frequency jet ventilation (HFJV). *Study Design*. Retrospective chart review was performed of infants with antenatal diagnosis of CDH born between 2004 and 2009, at Mount Sinai Hospital Toronto, Ontario, Canada. Detailed information was abstracted from the charts of all infants who received HFJV. *Results*. Of the 55 infants, 25 were managed with HFJV at some point during resuscitation and stabilization prior to transport. HFJV was the initial ventilation mode in six cases and nineteen infants were placed on HFJV as rescue therapy. Blood gases procured from the umbilical artery before and/or after the initiation of HFJV. There was a significant difference detected for both PaCO_2_ (*P* = 0.0002) and pH (*P* < 0.0001). The pre- and posttransport vital signs remained stable and no transport related deaths or significant complications occurred. *Conclusion*. HFJV appears to be safe and effective providing high frequency rescue therapy for infants with CDH failing conventional mechanical ventilation. This paper supports the decision to utilize HFJV as it likely contributed to safe transport of many infants that would not otherwise have tolerated transport to a surgical centre.

## 1. Introduction 

Congenital diaphragmatic hernia (CDH) is one of the most challenging malformations that neonatologists and pediatric surgeons must manage [1, 2]. In patients with antenatally diagnosed CDH, the prognosis is dependent on both the degree of lung hypoplasia and persistent pulmonary hypertension (PPHN) after their birth [1, 3]. The care of these infants has seen significant evolution, from previous aggressive ventilation and emergent surgical repair to current physiologic stabilization, standardized management protocols, gentle ventilation strategies, and delayed surgical repair, all in less than two decades [2]. 

Survival of patients with CDH is dependent on early diagnosis and improved resuscitation and transportation of an optimally-supported baby to a major surgical center for repair [3–5]. Infants with CDH may be diagnosed antenatally and deliver at a high risk perinatal center. Most infants with CDH require respiratory support with set limits on ventilatory pressures to avoid lung overdistension and acceptance of adequate rather than optimal PaCO_2_ and PaO_2_ [1]. High-frequency ventilation (HFV) allows gas exchange at low volumes thereby decreasing iatrogenic pulmonary barotrauma [6]. To date two modes of HFV has been studied in the care of infants with CDH: high frequency oscillatory ventilation (HFOV) and high frequency jet ventilation (HFJV). Careful use of HFV, either empirically or as rescue in infants requiring high peak inspiratory pressure (PIP) with conventional mechanical ventilation, appears to reduce mortality [7]. The use of HFV is well described in infants with CDH [8, 9]. However, transport with the HFO (3100A Sensormedics) is not an option as it does not have external battery power. HFJV is another mode of HFV which has previously been shown to be lung protective and can be used for transport as it has an external battery.

There is little published that describes the use HFJV during stabilization and transport of these infants [10]. The objective of this study was to review our site's experience with the transport and stabilization of infants with CDH who were treated with HFJV. Special considerations for managing an infant on HFJV during transport were also identified.

## 2. Methods

Following approval of the project by the Research Ethics Board of Mount Sinai Hospital, a retrospective chart review was performed of all infants with an antenatal diagnosis of CDH who were delivered at Mount Sinai Hospital, between January 01, 2004 and December 31, 2009. Mount Sinai Hospital (MSH) is a perinatal referral centre for fetal anomalies in the province of Ontario, Canada, has a level 3 NICU and adjoins The Hospital for Sick Children (HSC), a pediatric surgical site, through an underground tunnel. The records of all liveborn infants with congenital diaphragmatic hernia were identified. Data was abstracted from records of the resuscitation, stabilization and transport of these infants in addition to the records at the surgical site. The parameters reviewed included the initial steps taken during resuscitation, initial ventilation settings, and acid-base balance. The criteria for starting HFJV, pharmacological support required, time and length of transport, and any complications that arose in transit were also reviewed. 

Mount Sinai Hospital uses a standardized protocol for the resuscitation, and stabilization of these infants. Specifically, there are guidelines which limit the use of high airway pressures during conventional ventilation. Mean airway pressures (MAP) ≤ 12 cm H_2_O and PIP ≤ 25 cm H_2_O are targeted. Also, preductal saturation ≥85% is accepted, as well as tolerating mild-to-moderate hypercarbia (PaCO_2_ ≥ 55 mmHg, pH > 7.25). As this is considered a nonrecruitable lung disease, the use of HFV is recommended to optimize oxygenation while improving CO_2_ elimination. Nitric Oxide (20 ppm) is initiated when the oxygen index is more than 20 or at the discretion of the attending physician, and PGE_1_ infusion or inotropic agents were started at the discretion of the clinical team. The transport team consisted of a fellow (paediatrician in training to be a neonatologist), one or two registered nurses, and two respiratory therapists. 

An Airborne brand portable transport incubator (International Biomedical, USA) was used for all transports and modified to accommodate HFJV within this system. The Life Pulse high-frequency jet ventilator (Bunnell Inc, Salt Lake City, Utah) was used in tandem with a conventional ventilator to provide a source of heated, humidified bias flow. The iNOvent was used to deliver iNO which was blended into the constant flow of gas delivered through the HFJV circuit. The resuscitation team, under the direction of the attending neonatologist, determined the need to change from conventional mechanical ventilation to HFJV based on guidelines for a protective lung strategy as presented above. Variables were recorded before and after changing the ventilator, shortly prior to departure from the referring hospital, and upon arrival and stabilization in surgical center. Data recorded as before and after were analyzed by paired *t*-test. Significance was defined as *P* < 0.05. 

## 3. Results

Fifty-five infants were born with antenatally diagnosed CDH during the 6-year-study period. HFJV was used at some point in the management of 25 of those infants, which is 45% of all the CDH births.  Table 1 shows the data of demographics of CDH group. Sixteen were male and nine were female which corresponds to nearly a 2 : 1 male predominance. Ten infants (40%) had congenital anomalies other than CDH, 9 (36%) infants had known or suspected congenital heart disease, one with micrognathia and small kidneys, and one with bilateral pelviectasis also had a cardiac defect. Seven of the infants were born preterm (gestational age <36 + 6 weeks). 

Table 1 also presents the data on predictors of survival, observed/expected lung-to-head ratio (O/E LHR), and the predicted survival rate based on the regression equation of the CDH study group. At the time of this study neither of these predictors was used to guide therapy in an individual patient. 

The decision made to start a patient on high frequency ventilation was based on the clinical condition of the patient but in some patients was anticipated based on the severity of the infant's ultrasound findings. HFJV was the initial mode of ventilation used in six cases. In the remaining nineteen infants, HFJV was used as a rescue therapy for severe respiratory acidosis or hypoxemia despite maximal conventional ventilation parameters. One infant was transported on conventional ventilation because a provincial transport service was utilized that did not offer HFJV. The other infant was put back to conventional ventilation because of difficulties managing the infant on HFJV with secretions. 

Seven of the 25 infants died in the delivery site prior to transport. Of the seven infants who died soon after birth, one had a bilateral CDH and cardiac disease, two had right-sided hernias (one with cardiac disease), and four had left-sided hernias (two with multiple anomalies and one with cardiac disease). The remaining 18 were transferred to HSC, two on conventional ventilation and 16 on HFJV. 

Mount Sinai Hospital follows a protocol for managing CDH that defines failure of conventional ventilation as respiratory acidosis or hypoxemia despite PIP > 25 cm H_2_O or MAP > 12 cm H_2_O. Two of the 25 infants developed a pneumomediastinum and four had pneumothoraces, three of whom died prior to transport. 

**Figure 1:**
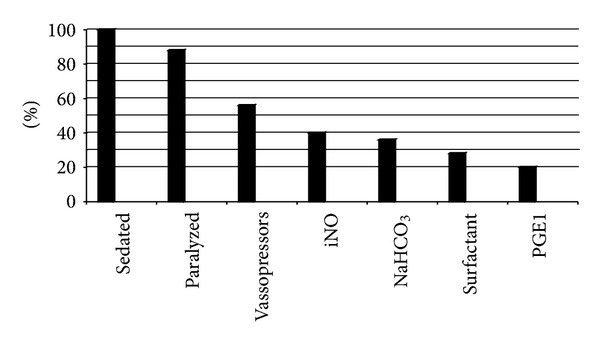
Management and interventions for all patient with CDH on HFJV.

Prior to transport all of the infants were sedated with either morphine or fentanyl and 88% (22/25) were paralyzed with Pavulon. Ten infants were transported on iNO. Seven received surfactant and five were treated with PGE_1_, seen in Figure 1.

**Figure 2:**
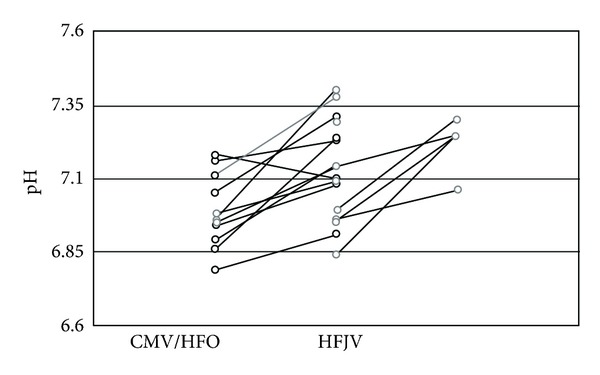
pH of patients before and after transition CMV or HFO to HFJV.

Twenty one of the infants had at least two blood gases procured from the umbilical artery before and/or after the initiation of HFJV and were found to have significant improvement in blood gases. Figure 2 illustrates the pH changes pre- and postinitiation of HFJV for each infant. All but one infant showed an improvement in pH; however, this infant actually experienced a decrease in PaCO_2_ and maintained a persistent metabolic acidosis. 

**Figure 3:**
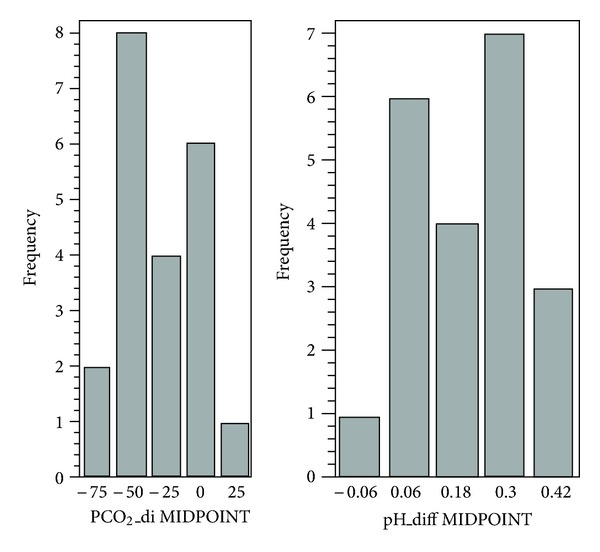
For the comparison of pre- versus post-PaCO_2_ and pH measurements, paired *t*-test was used. There was significant difference detected for both PCO_2_ (*P* = 0.0002) and pH (*P* < 0.0001).

For the comparison of pre- and postmeasurements of PaCO_2_ and pH, a paired *t*-test was used (Figure 3). There was a significant difference detected for both PaCO_2_ (*P* = 0.0002) and pH (*P* < 0.0001). 

The pre- and posttransport vital signs remained stable and no transport related deaths or significant patient complications occurred. The average length of transport from one site to the other was 30 minutes. The average time to rescue HFJV was 2 hours 14 min. The median time of birth to time of arrival to HSC was 5 hours 20 min. 

Data was collected from the Hospital for Sick Children on the sixteen patients transported using HFJV. Ten out of sixteen patients had the CDH surgically repaired, while care was withdrawn on six of the patients prior to surgery. Three of those infants had been treated with ECMO prior to their demise. The average age at time of surgery was 250.5 hrs (10.44 days). Eight patients had patch repairs, one had a primary repair and one had a thorascopic repair. Out of the 10 surgically repaired patients, 3 did not survive to discharge. One had a recurrence of the CDH while on ECMO and did not undergo a second surgery. Care was withdrawn on the others on post-op day 13 and 18, respectively, because of respiratory failure. There were seven survivors of the 16 patients transported to HSC on HFJV. The average length of stay in the ICU prior to discharge was 37.14 days. The overall mortality for this entire series was therefore 64% (16/25) despite maximal therapy including ECMO at the surgical site. 

## 4. Discussion

In most patients with CDH, respiratory management is complicated by the presence of multiple pathophysiologic challenges including hypoplastic and immature lungs and maldeveloped pulmonary vasculature [1–3]. The major advantage of high-frequency ventilation is improved oxygenation and ventilation through the use of small tidal volumes. Increased use of high-frequency ventilation, along with nitric oxide therapy, has decreased the need for ECMO in some centers [3–5]. HFJV has also been found to be effective in improving oxygenation and ventilation of neonates with CDH [11]. 

Our case series indicated that infants with CDH demonstrated significant improvement in ventilation upon initiation of HFJV and were able to be safely transported to a surgical hospital. These findings corroborate previous reports on the usefulness of HFJV as a rescue ventilation mode for neonates with respiratory failure supported by CMV [12, 13]. The first, Boros et al., describes the use of HFJV on five patients reporting one survivor. While, Kuluz et al., comments on the use of HFJV as rescue therapy in 16 patients with a predicted survival rate of 63%. The reported survival to discharge was 87%, supporting the possible usefulness of this therapy. 

Unlike most studies, this case series includes data on all infants with CDH without exclusion for LBW, prematurity, or congenital anomalies, yet they too experienced significant improvement in ventilation. Many different predictors of survival have been reported including the O/E lung to head ratio and the CDH study group predictive equation. Both have been validated in infants with isolated CDH [14, 15]. The predicted survival of this case series using either the O/E LHR or the CDH study group predictive equation was approximately 60% but our survival was 36%. The fact that more than half the infants, even those transported to the surgical site, did not survive to discharge, speaks to the severity of the illness and confirms the belief that it is indeed the most compromised infants that require HFJV in order to get to a surgical centre. 

Pneumothoraces dominated the complications in our study; however diagnostic imaging was performed after HFJV was initiated so it is difficult to assess the timing of the insult and whether it was related to the change in mode of ventilation. Indeed, the decision to change from CMV or HFOV to HFJV before transport was at times due to the recognition of the inability of CMV to achieve acceptable oxygenation and ventilation at lower pressures in the presence of an air leak. All of the cases of pneumothorax occurred prior to transport and were not a complication of the transport itself and none of the neonates with continuing air leak were unstable during transport. 

The use of HFV is well described in infants with CDH, however HFOV (3100A Sensormedics) cannot be used for transport due to lack of an external battery. Therefore, our center used HFJV to ventilate infants who require HFV in order to facilitate ground transport. The staff of MSH NICU have a high comfort level with this mode of ventilation and its use for transport. Our success in safely transporting this compromised population is due in part to this. Other centers unfamiliar or inexperienced with HFJV certainly would not achieve the same results. More recently other forms of HFOV are available that can be used in transport. A randomized controlled multicenter trial may be warranted to clarify the role of HFJV versus HFOV on the long-term outcomes of CDH.

Since this study is a retrospective analysis, there was no randomized assignment of ventilators (CMV or HFJV). This potential selection bias was mitigated by the fact that the sickest infants were placed on HFJV and yet it was these neonates that experienced a marked improvement. Despite the evidence of short-term improvement the overall benefit and long-term morbidity of this therapy needs to be further studied. To that end we are currently reviewing the overall outcomes of all antenatally diagnosed CDH at our sites. We also acknowledge the limited ability of our retrospective study to discuss minor or rare complications or adverse events during transport. 

## 5. Conclusions

HFJV appears to be a safe and effective method of providing high frequency rescue therapy for infants with CDH failing conventional mechanical ventilation. Our data indicates that HFJV may be the preferred method of support for this subset of transported neonates due to its ability to optimize ventilation. HFJV can also be provided safely and efficaciously during transport. This paper supports the decision to utilize HFJV as it contributed to the safe transport of infants that may not otherwise have tolerated transport to a surgical centre. Clearly, a prospective randomized controlled, multicenter study would be needed to affirm these findings.

## Figures and Tables

**Table 1:** Patient characteristics.

Male *n* (%)	16 (64%)
Birth weight grams mean (sd)	2868 (820)
Gestational age weeks mean (sd)	37.2 (3.3)
Left sided *n* (%)	19 (78%)
Bilateral *n* (%)	1 (4%)
Congenital anomalies *n* (%)	10 (40%)
Preterm *n* (%)	4 (16%)
*O/E LHR mean (sd)	39.3 (17.2)
**Probability for survival mean (sd)	0.6 (0.3)

*Observed/Expected lung-to-head ratio.

**Predicted survival based CDH study Group predictive equation.
